# Dose evaluation of inter- and intra-fraction prostate motion in extremely hypofractionated intensity-modulated proton therapy for prostate cancer

**DOI:** 10.1016/j.phro.2023.100474

**Published:** 2023-07-22

**Authors:** Sen-Quan Feng, Charlotte L. Brouwer, Erik W. Korevaar, Neha Vapiwala, Ken Kang-Hsin Wang, Curtiland Deville, Johannes A. Langendijk, Stefan Both, Shafak Aluwini

**Affiliations:** a Department of Radiation Oncology, University Medical Center Groningen, University of Groningen, Groningen, The Netherlands; b Department of Radiation Oncology, University of Pennsylvania, Philadelphia, PA, USA; c Biomedical Imaging and Radiation Technology Laboratory (BIRTLab), Department of Radiation Oncology, University of Texas Southwestern Medical Center, Dallas, TX, USA; d Department of Radiation Oncology and Molecular Radiation Sciences, Johns Hopkins University School of Medicine, Baltimore, MD, USA

**Keywords:** Intensity-modulated proton therapy, Extreme hypofractionation, Ultra-hypofractionation, Stereotactic body proton therapy, Prostate inter- and intra-fraction motion

## Abstract

Inter- and intra-fractional prostate motion can deteriorate the dose distribution in extremely hypofractionated intensity-modulated proton therapy. We used verification CTs and prostate motion data calculated from 1024 intra-fractional prostate motion records to develop a voxel-wise based 4-dimensional method, which had a time resolution of 1 s, to assess the dose impact of prostate motion. An example of 100 fractional simulations revealed that motion had minimal impact on planning dose, the accumulated dose in 95 % of the scenarios fulfilled the clinical goals for target coverage (D95 > 37.5 Gy). This method can serve as a complementary measure in clinical setting to guarantee plan quality.

## Highlights

• Synthetic computed tomography images were used to simulate real-time prostate position
• A 4D simulation method of intra-fractional prostate motion was developed
• Prostate motion had minimal dose impact to robustly optimized proton therapy

## 1 Introduction

External beam radiation therapy (EBRT) with photons using mild to moderate hypofractionation is increasingly utilized for patients with high-risk prostate carcinoma (PCa), in whom a lower local control rate and overall survival rate are still reported after definitive-intent treatment compared to low- and intermediate-risk PCa patients [1], [2], [3], [4], [5], [6], [7]. Due to the low alpha/beta ratio of PCa, dose escalation with extreme (or ultra-) hypofractionation schemes may theoretically improve local control and reduce treatment time. However, extreme hypofractionation may increase the dose to organs at risk (OARs) and thus increase the incidence of toxicities [8], [9], [10].

Intensity-modulated proton therapy (IMPT) can potentially reduce radiation dose to OARs compared to traditional EBRT [11], [12], [13]. However, IMPT could be more sensitive to interplay effects due to the time structure (proton beam was chronologically delivered layer by layer) of the beam and inter- and intra-fractional prostate motion, which could decrease target dose coverage [14] resulting in decreased disease control, while increasing dose to OARs and potentially leading to greater toxicity. Therefore, the impact of interplay effects in an extremely hypofractionated intensity-modulated proton therapy (EHIMPT) is important and should be investigated while considering clinical introduction.

In current clinical proton therapy practice, treatment plans are robustly optimized and evaluated for setup and range errors [15]. Although this method is generally more suitable than planning target volume-based plan evaluation, it does not explicitly incorporate the dose impact of inter- and intra-fraction prostate motion during beam delivery.

Some IMPT simulation studies quantified the dose impact of prostate motion by using the prostate motion of individual patients or the worst-case motion scenario [16], [17], [18], [19]. However, a more common range of prostate motion was not investigated to provide evidence for common clinical practice since prostate motion does not have a predictable direction and magnitude [20]. Furthermore, earlier studies [21], [22] used the planning computed tomography (pCT) scan to simulate both inter- and intra-fraction prostate motion, neglecting anatomical variations between fractions.

This study aimed to develop a new method for evaluating the impact of inter- and intra-fraction prostate motion on the dose distribution during the delivery of a robustly optimized EHIMPT treatment. This was achieved by utilizing four-dimensional (4D) synthetic computed tomography (sCT) simulations. The method was applied to an example patient anatomy and EHIMPT plan.

## 2 Materials and methods

### 2.1 Patient

**Fig. 1:**
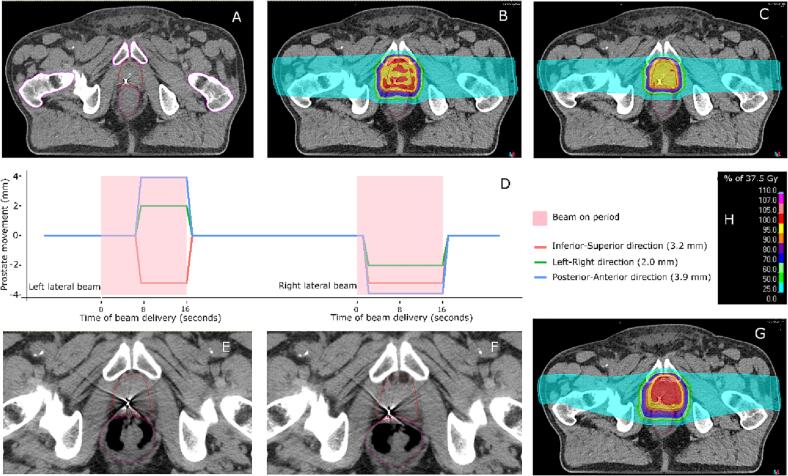
(A) Planning CT; (B) Nominal dose distribution; (C) Voxel-wise minimum dose distribution; (D) Example of one fraction with randomized prostate motion and timing; (E) Synthetic CT left lateral beam; (F) synthetic CT right lateral beam; (G) Simulated accumulated dose distribution planning CT; (H) Dose distribution legend. Solid lines delineate the targets’ and organs’ positions before movement, dotted lines delineate the targets’ and organs’ positions after movement.

This study used the CT imaging data (including pCT (Fig. 1A) and five weekly verification CT (vCT) images) of a representative PCa patient (within standardized follow-up program) treated with conventionally fractionated photon therapy (35 × 2.2 Gy). Structures including clinical target volume (CTV), CTV1 (prostate), CTV2 (proximal 2 cm of seminal vesicles) were delineated by an experienced radiation oncologist, and bladder, rectum, anal canal, femoral heads, rectal wall, and skin were delineated by an expert radiotherapy technician and checked by a radiation oncologist. The patient consented for data usage within standardized follow-up program (Medical Ethical Committee permission number: 518/2017).

### 2.2 Treatment planning

An EHIMPT plan (5 × 7.5 Gy, relative biological effectiveness = 1.1) for this patient was generated in RayStation Research 7.99 (RaySearch Laboratories, Stockholm, Sweden) according to our protocol constraints (Table 1). Every fraction consisted of two lateral beams (90°, 270°). Each beam contained 11 energy layers, delivered in a raster pattern [23]. A phantom (with fiducial markers) treatment plan delivery showed a delivery time for each beam of 16 s. The plan was robustly optimized using a 5 mm setup and 3 % range uncertainty. For evaluation purposes, each beam was split into 16 sub-plans, each representing the beam delivery for one second.

**Table 1:** Dose constraints and dose results of original plan dose and simulated accumulated dose.

	Dose constraints	Original plan dose (Gy)		Simulated accumulated dose (Gy)			
		Nominal dose	Voxel-wise Worst dose	Mean dose	95 % CI		Worst-case dose
**CTVs**							
CTV1	D95 % > 37.5 Gy	37.9	37.2	37.9	37.7	38.1	37.0
	HI	0.03	0.07	0.04	0.04	0.05	0.07
CTV2	D95 % > 30.0 Gy	30.3	29.2	31.6	31.5	31.8	31.1
	HI	0.26	0.24	0.21	0.20	0.21	0.23
**OARs**							
Anal Canal	D0.01 cm^3^ < 26.3 Gy	19.8	29.6	19.1	18.8	19.5	20.4
Anal Canal	D5 cm^3^ < 15.0 Gy	0.9	1.9	0.8	0.8	0.8	0.8
Bladder	D0.01 cm^3^ < 39.8 Gy	40.4	41.5	39.2	38.9	39.4	40.8
Bladder	D10 cm^3^ < 26.3 Gy	36.7	38.5	36.0	35.9	36.2	36.4
Left Femoral Head	D0.01 cm^3^ < 22.3 Gy	15.8	16.4	15.4	15.4	15.5	15.4
Left Femoral Head	D10 cm^3^ < 20.6 Gy	14.4	14.7	13.9	13.9	13.9	13.9
Right Femoral Head	D0.01 cm^3^ < 22.5 Gy	15.9	16.5	15.1	15.1	15.1	15.1
Right Femoral Head	D10 cm^3^ < 20.6 Gy	14.6	14.8	14.3	14.3	14.3	14.3
Rectum	D2 cm^3^ < 39.4 Gy	36.0	38.5	35.8	35.5	36.0	36.4
Rectum	D5 cm^3^ < 35.6 Gy	32.1	35.2	32.4	32.2	32.6	33.0
Anterior rectal wall	D0.01 cm^3^ < 39.4 Gy	39.4	41.1	39.2	39.0	39.4	40.1
Posterior rectal wall	D0.01 cm^3^ < 18.8 Gy	19.0	30.3	26.8	26.7	26.8	26.9
Skin	D0.01 cm^3^ < 15.0 Gy	14.3	14.5	13.1	13.0	13.1	13.1

### 2.3 Prostate motion data

Prostate motion data of twenty-six PCa patients treated with conventionally fractionated radiotherapy (without endorectal device) in the Department of Radiation Oncology of the University of Pennsylvania were used [24]. The prostate motion was recorded in real-time at a frequency of 10 Hz in three degrees of freedom using a 4D electromagnetic tracking system (Calypso Medical Technologies, Seattle, WA).

Among 1055 available treatment fractions’ records, all records longer than four-minute treatment time were selected (1024), since the time interval for every fractional treatment would be at least four minutes in our medical center. The 95th percentile of motion range was calculated in each dimension (left right (x), inferior superior (y), and posterior anterior (z)). Every range in each direction yields two opposite 1-dimensional vectors: (0, ±x), (0, ±y), (0, ±z). Equispaced sparse sampling resulted in eight 3-dimensional (3D) motion vectors: (x, y, z), (x, y, -z), (x, -y, z), (x, -y, -z), (-x, y, z), (-x, y, -z), (-x, -y, z), (-x, -y, -z).

Six common motion trajectories were reported by Kupelian et al. [25]. We chose to simulate the motion with a sudden change in order to observe an obvious dose change [19].

### 2.4 Synthetic CT

Five weekly vCTs were used to simulate five fractions of EHIMPT. Each vCT was registered to the pCT based on fiducial markers according to the institutional procedure for online verification.

Each weekly vCT was used to create eight sCTs based on the eight 3D motion vectors. The CTVs were translated within the patient according to the motion vector (x, y, z). Next, the CTVs and pelvic bones were set as controlling regions of interest (ROIs) for the hybrid deformable image registration (Anatomically constrained deformation algorithm (ANACONDA) in RayStation Research 7.99) to obtain the deformation vector fields (DVFs), and created a sCT (Fig. 1E, Fig. 1F) that illustrates the pelvic anatomy. These procedures were performed for all eight motion vectors on each of the 5 vCTs, resulting in 40 sCTs. Each sCT illustrated the anatomy of corresponding motion scenarios determined as described above. The OARs were delineated at each vCT and were deformed according to the obtained DVFs, so their intra-fraction motion was modeled by the deformation caused by the prostate motion.

### 2.5 Randomization of prostate motion vectors and timing

For each beam, a combination of motion vector and motion initiating time was randomly selected from the 128 combinations (8 motion vectors and 16 initiation time) with a time resolution of 1 s. We conducted 100 fractional simulations, with each motion scenario randomly selected using R (R, Vienna, Austria), to obtain 20 complete treatment simulations. Fig. 1D shows an example of one randomized motion.

### 2.6 Dose accumulation

For each EHIMPT, the sub-plan of every second was calculated on the corresponding sCT. Using the DVFs obtained by ANACONDA, all sub-plan doses on sCTs were warped back to the vCTs of every fraction, and then the five-fraction doses on vCTs were warped and summed to the pCT (Fig. A.1 and A.2 in supplement). The accumulated dose on the pCT (Fig. 1G) represents the simulated accumulated dose of one treatment. Accumulated doses from 20 simulated treatments were compared to the nominal dose (Fig. 1B) and the voxel-wise worst dose (Fig. 1C): voxel-wise minimal dose in CTVs and voxel-wise maximal dose in OARs [15].

## 3 Results

The 95th percentile of prostate motion range were 2.0 mm in lateral direction, 3.2 mm in inferior-superior direction, and 3.9 mm in posterior-anterior directions.

In the 20 simulated treatments, the mean simulated accumulated doses met the dose constraints for CTV1 and CTV2 with D95 % of 37.9 Gy [95 % CI 37.1 – 38.1] and 31.6 Gy [95 % CI 31.5 – 31.8] (Table 1). The worst-case dose (D95 %) to CTV1 in 20 simulated treatments was just below constraint (37.0 Gy).

When comparing the mean simulated accumulated dose to nominal dose of CTV1, no difference existed, with 37.9 Gy in the mean simulated accumulated dose and in the nominal dose. However, a difference is reported for D95 % dose of CTV2, with 31.6 Gy in the mean simulated accumulated dose versus 30.3 Gy in the nominal dose (Table 1).

Regarding OAR dose, the mean simulated accumulated dose was lower than the nominal dose in all dose constraints except D0.01 cm^3^ of the posterior rectal wall and D5cm^3^ of the rectum. The largest difference was found in the D0.01 cm^3^ of the posterior rectal wall with 26.8 Gy in the mean simulated accumulated dose and 19.0 Gy in nominal dose (Table 1).

## 4 Discussion

We presented a probability-based motion simulation method for evaluating inter- and intra-fraction motion in robustly optimized EHIMPT. Simulated accumulated doses to the target volumes were adequate in the patient anatomy investigated.

In our analysis we considered both the simulated accumulated dose and the voxel-wise worst dose. Voxel-wise worst doses were calculated without considering 4D prostate motion (but only patient setup and CT range errors). Simulations in this study used perfect setup during beam delivery, but included repeated imaging to include inter-fraction motion and synthetic imaging with a 4D resolution of 1 s to include intra-fraction motion. Therefore, our method can be used additional and complementary to the standard robustness evaluation method of proton plans using setup and range errors.

The probability-based motion simulation in our study is based on a randomization procedure applying a range of motion (95 % range derived from 1024 fractions of 26 patients). This was done to simulate a wider range of motion possibilities while excluding the extreme outliers. Other studies [21], [26] simulated selected motion trajectories that represent extreme motion scenarios, which was less representative. The most common prostate motion is a gradual drift, but it has minimal impact to the dose distribution [19]. We chose to simulate the motion trajectory of a sudden intra-fraction prostate motion to observe an obvious dose alteration [25].

In our study, the mean and the minimal dose (worst-case dose) of CTV1-D95 % (prostate) in simulated treatments were 37.9 Gy and 37.0 Gy which are 101.1 % and 98.7 % of the prescribed dose. This is comparable to the results of Su et al. (103.1 % (mean) and 96.7 % (worst-case minimal dose) using uniform scanning proton delivery technique) [21]. Our study showed a small dose variation of 2.4 % between mean and minimum dose, compared to 6.2 % reported by Su et al. which could be due to the use of 5 mm robustness margin compared to 4 mm by Su et al. Besides, simulation of prostate motion reported by Su et al. was less representative for the average PCa patients because of the use of up to 15 mm motion range in one fraction, which rarely occurred [27], [28], [29]. Ammazzalorso et al. [22] reported a median (worst) CTV dose drop of 0.5 % (2.8 %) in a 4D intra-fractional prostate motion simulation study, compared to a mean (worst) CTV dose drop of 1.1 % (1.3 %) in our study; the small difference may be caused by the compensation effect of fractionation.

A limitation is that generating sCTs based merely on rigid displacement of the prostate. This problem is inevitable since the Calypso data do not include organ deformation or rotational information. Rotations could result in target dose reduction [30], and severe organ deformation could occur when the prostate moves abruptly [31], [32]. At certain instance, the worst-case simulated accumulated dose was even lower than the nominal dose to OARs, a finding consistent with previous studies [16], [33]. This occurrence can be attributed to the combined impact of imperfect dose warp, inter-fractional anatomical variation, and the limited representation of deformed OARs in the sCT.

Our study was limited to simulating only one patient’s anatomy and treatment plan. However, the focus was on the implementation of the simulation method. The selected patient had the largest inter-fraction motion among all patients with available vCTs. For patients with possible larger prostate volumes, the amount of energy layers and delivery time increase accordingly (e.g. CTV1 volume increased to 54.9 cm^3^ needed 13 energy layers per beam).

We will be using the presented probability-based simulation method as an additional tool to the setup and range robustness evaluation of EHIMPT plans to gain more insight and confidence in the robustness of EHIMPT against inter- and intra-fraction prostate motion in a larger group of patients and treatment plans.

In conclusion, this study introduced a new, 4-D simulation method to assess the dose impact of inter- and intra-fraction motion. This method can be used in addition to the robust evaluation method.

## CRediT authorship contribution statement

**Sen-Quan Feng:** Conceptualization, Methodology, Software, Data curation, Writing – original draft, Writing – review & editing, Visualization. **Charlotte L. Brouwer:** Conceptualization, Methodology, Supervision, Writing – review & editing, Visualization, Project administration. **Erik W. Korevaar:** Methodology, Writing – review & editing. **Neha Vapiwala:** Resources, Data curation, Writing – review & editing. **Ken Kang-Hsin Wang:** Resources, Data curation, Writing – review & editing. **Curtiland Deville:** Resources, Data curation, Writing – review & editing. **Johannes A. Langendijk:** Conceptualization, Resources, Supervision, Writing – review & editing, Project administration, Funding acquisition. **Stefan Both:** Resources, Data curation, Writing – review & editing. **Shafak Aluwini:** Conceptualization, Resources, Methodology, Supervision, Writing – review & editing, Project administration, Funding acquisition.

## Declaration of Competing Interest

The authors declare that they have no known competing financial interests or personal relationships that could have appeared to influence the work reported in this paper.

## References

[1] Reese A.C., Pierorazio P.M., Han M., Partin A.W. (2012). Contemporary evaluation of the national comprehensive cancer network prostate cancer risk classification system. Urology.

[2] Joniau S., Briganti A., Gontero P., Gandaglia G., Tosco L., Fieuws S. (2015). Stratification of high-risk prostate cancer into prognostic categories: A european multi-institutional study. Eur Urol.

[3] Kuban D.A., Tucker S.L., Dong L., Starkschall G., Huang E.H., Cheung M.R. (2008). Long-Term Results of the M. D. Anderson Randomized Dose-Escalation Trial for Prostate Cancer. Int J Radiat Oncol Biol Phys.

[4] Heemsbergen W.D., Al-Mamgani A., Slot A., Dielwart M.F.H., Lebesque J.V. (2014). Long-term results of the Dutch randomized prostate cancer trial: Impact of dose-escalation on local, biochemical, clinical failure, and survival. Radiother Oncol.

[5] Zietman A.L., Bae K., Slater J.D., Shipley W.U., Efstathiou J.A., Coen J.J. (2010). Randomized trial comparing conventional-dose with high-dose conformal radiation therapy in early-stage adenocarcinoma of the prostate: Long-term results from Proton Radiation Oncology Group/American College Of Radiology 95–09. J Clin Oncol.

[6] Beckendorf V., Guerif S., Le Prisé E., Cosset J.M., Bougnoux A., Chauvet B. (2011). 70 Gy versus 80 Gy in localized prostate cancer: 5-year results of GETUG 06 randomized trial. Int J Radiat Oncol Biol Phys.

[7] Dearnaley D.P., Jovic G., Syndikus I., Khoo V., Cowan R.A., Graham J.D. (2014). Escalated-dose versus control-dose conformal radiotherapy for prostate cancer: Long-term results from the MRC RT01 randomised controlled trial. Lancet Oncol.

[8] Fransson P., Nilsson P., Gunnlaugsson A., Beckman L., Tavelin B., Norman D. (2021). Ultra-hypofractionated versus conventionally fractionated radiotherapy for prostate cancer (HYPO-RT-PC): patient-reported quality-of-life outcomes of a randomised, controlled, non-inferiority, phase 3 trial. Lancet Oncol.

[9] Yu J.B., Cramer L.D., Herrin J., Soulos P.R., Potosky A.L., Gross C.P. (2014). Stereotactic body radiation therapy versus intensity-modulated radiation therapy for prostate cancer: Comparison of toxicity. J Clin Oncol.

[10] Kishan A.U., King C.R. (2017). Stereotactic Body Radiotherapy for Low- and Intermediate-Risk Prostate Cancer. Semin Radiat Oncol.

[11] Hammer C., Brouwer C.L., Klinker P., Both S., Aluwini S., Langendijk J.A. (2018). PO-0821: Radiation-induced late rectal toxicity for IMPT vs VMAT in patients with localized prostate cancer. Radiother Oncol.

[12] Dowdell S.J., Metcalfe P.E., Morales J.E., Jackson M., Rosenfeld A.B. (2008). A comparison of proton therapy and IMRT treatment plans for prostate radiotherapy. Australas Phys Eng Sci Med.

[13] Vargas C., Fryer A., Mahajan C., Indelicato D., Horne D., Chellini A. (2008). Dose-Volume Comparison of Proton Therapy and Intensity-Modulated Radiotherapy for Prostate Cancer. Int J Radiat Oncol Biol Phys.

[14] Nejad-Davarani S.P., Sevak P., Moncion M., Garbarino K., Weiss S., Kim J. (2019). Geometric and dosimetric impact of anatomical changes for MR-only radiation therapy for the prostate. J Appl Clin Med Phys.

[15] Korevaar E.W., Habraken S.J.M., Scandurra D., Kierkels R.G.J., Unipan M., Eenink M.G.C. (2019). Practical robustness evaluation in radiotherapy – A photon and proton-proof alternative to PTV-based plan evaluation. Radiother Oncol.

[16] Tang S., Deville C., Tochner Z., Wang K.K.H., McDonough J., Vapiwala N. (2014). Impact of intrafraction and residual interfraction effect on prostate proton pencil beam scanning. Int J Radiat Oncol Biol Phys.

[17] Thörnqvist S., Muren L.P., Bentzen L., Hysing L.B., Hoyer M., Grau C. (2013). Degradation of target coverage due to inter-fraction motion during intensity-modulated proton therapy of prostate and elective targets. Acta Oncol.

[18] Moteabbed M., Trofimov A., Sharp G.C., Wang Y., Zietman A.L., Efstathiou J.A. (2016). A Prospective Comparison of the Effects of Interfractional Variations on Proton Therapy and Intensity Modulated Radiation Therapy for Prostate Cancer. Int J Radiat Oncol Biol Phys.

[19] Ammazzalorso F., Graef S., Weber U., Wittig A., Engenhart-Cabillic R., Jelen U. (2014). Dosimetric consequences of intrafraction prostate motion in scanned ion beam radiotherapy. Radiother Oncol.

[20] Ballhausen H., Li M., Hegemann N.S., Ganswindt U., Belka C. (2015). Intra-fraction motion of the prostate is a random walk. Phys Med Biol.

[21] Su Z., Slopsema R., Flampouri S., Li Z. (2019). Impact of intrafraction prostate motion on clinical target coverage in proton therapy: A simulation study of dosimetric differences in two delivery techniques. J Appl Clin Med Phys.

[22] Ammazzalorso F., Jelen U.A. (2014). 4D dose computation method to investigate motion interplay effects in scanned ion beam prostate therapy. Phys Med Biol.

[23] Trofimov A., Bortfeld T. (2003). Optimization of Beam Parameters and Treatment Planning for Intensity Modulated Proton Therapy. Technol Cancer Res Treat.

[24] Wang K.K.H., Vapiwala N., Deville C., Plastaras J.P., Scheuermann R., Lin H. (2012). A study to quantify the effectiveness of daily endorectal balloon for prostate intrafraction motion management. Int J Radiat Oncol Biol Phys.

[25] Kupelian P., Willoughby T., Mahadevan A., Djemil T., Weinstein G., Jani S. (2007). Multi-institutional clinical experience with the Calypso System in localization and continuous, real-time monitoring of the prostate gland during external radiotherapy. Int J Radiat Oncol Biol Phys.

[26] Tang S., Deville C., McDonough J., Tochner Z., Wang K.K.H., Vapiwala N. (2013). Effect of intrafraction prostate motion on proton pencil beam scanning delivery: A quantitative assessment. Int J Radiat Oncol Biol Phys.

[27] Tong X., Chen X., Li J., Xu Q., Lin M., han,, Chen, L, (2015). Intrafractional prostate motion during external beam radiotherapy monitored by a real-time target localization system. J Appl Clin Med Phys.

[28] Both S., Wang K.K.H., Plastaras J.P., Deville C., Bar A.V., Tochner Z. (2011). Real-time study of prostate intrafraction motion during external beam radiotherapy with daily endorectal balloon. Int J Radiat Oncol Biol Phys.

[29] Juneja P., Kneebone A., Booth J.T., Thwaites D.I., Kaur R., Colvill E. (2015). Prostate motion during radiotherapy of prostate cancer patients with and without application of a hydrogel spacer: A comparative study. Radiat Oncol.

[30] Amro H., Hamstra D.A., McShan D.L., Sandler H., Vineberg K., Hadley S. (2013). The dosimetric impact of prostate rotations during electromagnetically guided external-beam radiation therapy. Int J Radiat Oncol Biol Phys.

[31] Ghilezan M.J., Jaffray D.A., Siewerdsen J.H., Van Herk M., Shetty A., Sharpe M.B. (2005). Prostate gland motion assessed with cine-magnetic resonance imaging (cine-MRI). Int J Radiat Oncol Biol Phys.

[32] Hegde J.V., Cao M., Yu V.Y., Kishan A.U., Shaverdian N., Lamb J. (2018). Magnetic Resonance Imaging Guidance Mitigates the Effects of Intrafraction Prostate Motion During Stereotactic Body Radiotherapy for Prostate Cancer. Cureus.

[33] D.M. De Muinck Keizer C. Kontaxis Kerkmeijer LGW,Van Der Voort Van Zyp JRN, Van Den Berg CAT, Raaymakers BW, et al. Dosimetric impact of soft-tissue based intrafraction motion from 3D cine-MR in prostate SBRT Phys Med Biol 65 2020 10.1088/1361-6560/ab6241. 10.1088/1361-6560/ab6241 31842008

