# Supplementary material for: Dose evaluation of inter- and intra-fraction prostate motion in extremely hypofractionated intensity-modulated proton therapy for prostate cancer
Source: Phys Imaging Radiat Oncol. 2023 Jul 22;27:100474. doi: 10.1016/j.phro.2023.100474 (PMC10407426; doi:10.1016/j.phro.2023.100474)
Supplement: Supplementary data 1 [file mmc1.docx]

Supplementary material


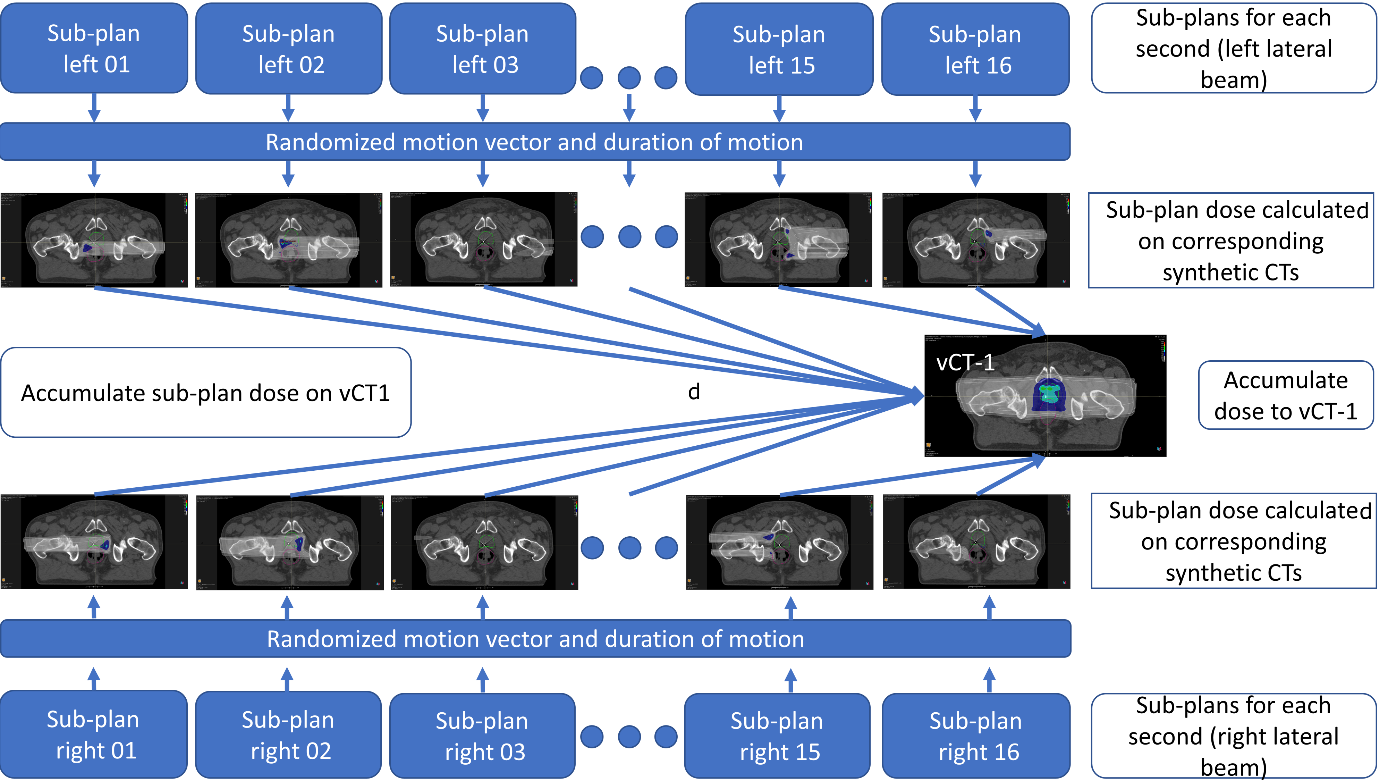


Fig. A.1. Sub-plans dose accumulate and sum to vCT. vCT: verification CT. This process is repeated for vCT-2, 3, 4 and 5.


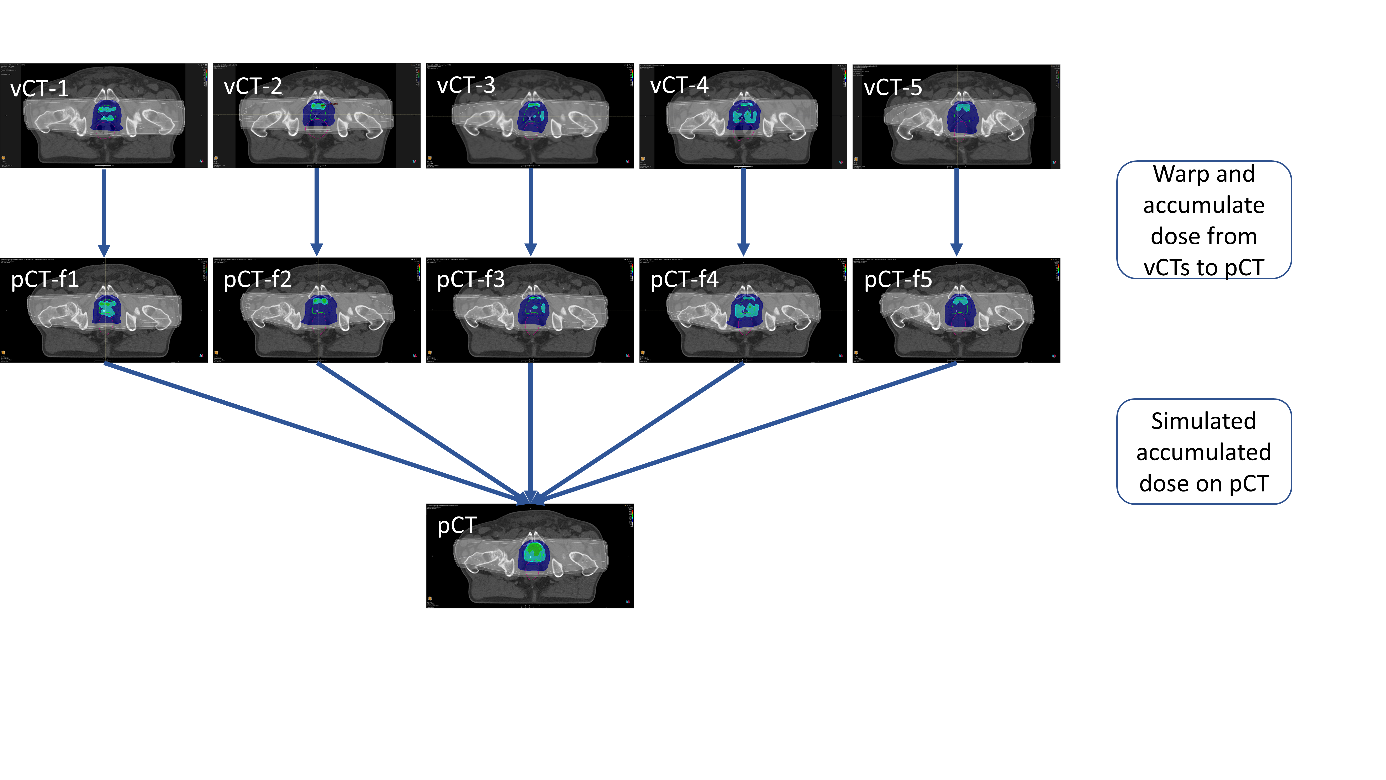


Fig. A.2. Dose accumulation and sum from vCT to pCT. pCT: planning CT; vCT: verification CT, f1:fraction 1, f2: fraction 2, f3: fraction 3, f4: fraction 4, f5: fraction 5.
